# Gastric cancer at a university teaching hospital in northwestern Tanzania: a retrospective review of 232 cases

**DOI:** 10.1186/1477-7819-10-257

**Published:** 2012-11-27

**Authors:** Joseph B Mabula, Mabula D Mchembe, Mheta Koy, Phillipo L Chalya, Fabian Massaga, Peter F Rambau, Nestory Masalu, Hyasinta Jaka

**Affiliations:** 1 Department of Surgery, Catholic University of Health and Allied Sciences-Bugando, Mwanza, Tanzania; 2 Department of Surgery, Muhimbili University of Health and Allied Sciences, Dar Es Salaam, Tanzania; 3 Department of Internal Medicine, Catholic University of Health and Allied Sciences-Bugando, Mwanza, Tanzania; 4 Department of Surgery, University of Dodoma, Dodoma, Tanzania; 5 Department of Pathology, Catholic University of Health and Allied Sciences-Bugando, Mwanza, Tanzania; 6 Department of Oncology, Catholic University of Health and Allied Sciences-Bugando, Mwanza, Tanzania

**Keywords:** Gastric cancer, Clinicopathological pattern, Treatment outcome, Tanzania

## Abstract

**Background:**

Despite marked decreases in its incidence, particularly in developed countries, gastric cancer is still the second most common tumor worldwide. There is a paucity of information regarding gastric cancer in northwestern Tanzania. This study was undertaken to describe our experience, in our local setting, on the management of gastric cancer, outlining the clinicopathological and treatment outcome of these patients and suggesting ways to improve the treatment outcome.

**Methods:**

This was a retrospective study of histologically confirmed cases of gastric cancer seen at Bugando Medical Centre between January 2007 and December 2011. Data were retrieved from patients’ files and analyzed using SPSS computer software version 17.0.

**Results:**

A total of 232 gastric cancer patients were enrolled in the study, representing 4.5% of all malignancies. The male to female ratio was 2.9:1. The median age of patients was 52 years. The majority of the patients (92.1%) presented late with advanced gastric cancer (Stages III and IV). Lymph node and distant metastasis at the time of diagnosis was recorded in 31.9% and 29.3% of cases, respectively. The antrum was the most frequent anatomical site (56.5%) involved and gastric adenocarcinoma (95.1%) was the most common histopathological type. Out of 232 patients, 223 (96.1%) patients underwent surgical procedures for gastric cancer of which gastro-jejunostomy was the most frequent performed surgical procedure, accounting for 53.8% of cases. The use of chemotherapy and radiotherapy was documented in 56 (24.1%) and 12 (5.1%) patients, respectively. Postoperative complication and mortality rates were 37.1% and 18.1%, respectively. According to multivariate logistic regression analysis, preoperative co-morbidity, histological grade and stage of the tumor, presence of metastases at the time of diagnosis was the main predictors of death (*P* <0.001). At the end of five years, only 76 (32.8%) patients were available for follow-up and the overall five-year survival rate was 6.9%. Evidence of cancer recurrence was reported in 45 (19.4%) patients. Positive resection margins, stage of the tumor and presence of metastasis at the time of diagnosis were the main predictors of local recurrence (*P* <0.001).

**Conclusions:**

Gastric cancer in this region shows a trend towards relative young age at diagnosis and the majority of patients present late with an advanced stage. Lack of awareness of the disease, poor accessibility to health care facilities and lack of screening programs in this region may contribute to advanced disease at the time of diagnosis. There is a need for early detection, adequate treatment and proper follow-up to improve treatment outcome.

## Background

Gastric cancer is one of the most common cancers and remains a major public health problem as the fourth most common cancer and the second leading cause of cancer death worldwide [1]. According to a global estimation, approximately 985,600 new cases of gastric cancer are being diagnosed each year and a minimum of 738,000 patients die from the disease [1,2]. Nearly two-thirds of gastric cancer occurs in the developing countries [3]. There is a wide variation in the incidence of gastric cancer in different geographical regions. While the incidence of gastric cancers is high in China, Japan and Korea, the incidence is comparatively low in most of Europe, North America and Africa [3]. Within the African continent, there are also great variations [2,4]. Nigeria and South Africa have been noted to have the highest incidence in Africa compared to West African nations, Egypt and Kenya [5]. Regional variations have also been observed within those countries. In Nigeria, the rate in the southwestern areas has been put at 4.1%, and double this rate in the northern part of the country [6,7]. In Tanzania, high incidence of gastric cancer has been reported in areas around Mount Kilimanjaro [8]. Differences in dietary and genetic factors and variation in the infectivity rate of *Helicobacter pylori* have been reported to be responsible for this variation [7,9].

The incidence of gastric cancer by tumor location has also been reported to vary widely based on geographic location, race and socioeconomic status [10]. Distal gastric cancer predominates in developing countries, among blacks, and in lower socioeconomic groups, whereas proximal tumors are more common in developed countries, among whites, and in higher socio-economic classes [11]. Diverging trends in the incidence of gastric cancer by tumor location suggest that they may represent two diseases with different etiologies. The main risk factors for distal gastric cancer include *H. pylori* infection and dietary factors; whereas, gastroesophageal reflux disease and obesity play important roles in the development of proximal stomach cancer [11,12].

Gastric cancer is a multifactorial disease involving both genetic and environmental factors [12]. Several factors are implicated in the development of gastric cancer, including diet, *H. pylori* infection, previous gastric surgery, pernicious anemia, adenomatous polyps, chronic atrophic gastritis, prior radiation exposure and genetic factors [12,13].

Gastric cancer is difficult to diagnose early because there is usually a time lag between the onset of growth and the appearance of symptoms [14]. Early symptoms of gastric cancer are non-specific and vague; as a result, most patients with early gastric cancer present with symptoms indistinguishable from benign peptic ulcer disease and, subsequently, these patients are diagnosed with late stage gastric cancer or one of its complications [7,15]. In low income countries like Tanzania, patients with dyspeptic symptoms are managed solely based on clinical examination because radiological and upper gastrointestinal endoscopic facilities are not available in many centers [7,8,13-15]. These patients present later with advanced gastric cancer, which may be unresectable, or relapse after complete resection. The detection of gastric cancer in the early stage is vitally important in ensuring an excellent prognosis. In Japan, up to 60% of gastric cancers are diagnosed as early cancers [15]. In developing countries, however, early disease is much less frequently detected due to poor endoscopic facilities and lack of mass screening programs [16]. Screening for this group of patients improves detection rate of early gastric cancer and, therefore, its prognosis. Endoscopy for surveillance of premalignant lesions has been explored with this objective in mind [15,17]. A high index of suspicion by clinicians, health education for early presentation and availability of endoscopic facilities may help to facilitate early diagnosis and improve outcome.

The management of gastric cancer presents a great challenge in resource limited countries as found in Africa. Late presentation of the disease, lack of adequate screening programs, lack of endoscopic facilities, lack of adjuvant therapy, and a high morbidity and mortality are among the hallmarks of the disease in these countries [16]. The treatment of gastric cancer depends on several factors, including the size, location and extent of the tumor, the stage of disease, the patient’s age and overall health [18]. Current treatment options of gastric cancer include surgery, chemotherapy and palliative care. Surgery is currently the only treatment option for gastric cancer with curative potential [18,19]. However, curative surgery in most developing countries is limited due to the late presentation in the majority of patients.

The clinical stage of the disease at presentation is important for the outcome of the patient with gastric cancer. However, the outcome of treatment of gastric cancer in our environment has been poor because the majority of these patients present late to the hospital with an advanced stage and only palliative care is possible [19]. This is partly due to the paucity of local data regarding this condition and lack of community awareness on the importance of early reporting to hospital for early diagnosis and treatment. The staging of the patient can be used to determine the prognosis of the patient. Whereas stage I disease has about a 60 to 80% five-year survival, this can be as low as a less than 5% five-year survival rate in stage IV [13].

The aim of this study was to describe our experience in our local setting on the management of gastric cancer, outlining the clinicopathological and treatment outcome of these patients and suggesting ways to improve treatment outcome.

## Methods

This was a retrospective study of patients with histological diagnosis of gastric cancer seen at the surgery department of Bugando Medical Centre (BMC) between January 2007 and December 2011. BMC is a consultant, tertiary care and teaching hospital for the Catholic University of Health and allied Sciences-Bugando (CUHAS-Bugando) and has a bed capacity of 1,000. It serves as a referral center for tertiary specialist care for a catchment population of approximately 13 million people. The hospital has a newly established oncology department which provides care for all patients with histopathologically-proven cancers, including gastric cancers. However, the department does not provide radiotherapy services at the moment due to lack of this facility at our center. As a result, patients requiring this modality of treatment have to travel long distances to receive radiotherapy at the Tanzania Tumor Centre located a considerable distance from the study area.

The subjects of this study included all patients who presented to BMC with histologically-confirmed gastric cancer during the period studied. Patients with incomplete data were excluded from the study.

The details of patients were retrieved from patients’ files kept in the medical record department, the surgical wards, operating theater and histopathology laboratory. Information retrieved included socio-demographic data, clinical presentation, anatomical site, gross appearance, TNM stage, histopathological type and grade, presence of metastasis (nodal, distant and peritoneal), treatment modalities, and outcome and follow-up. The clinical presentation was conveniently grouped into five categories according to the Uganda Cancer Working Group for gastric cancer diagnosis and treatment guidelines [20]. The clinical stage of the disease was assigned to each patient by using TNM (AJCC cancer staging manual); this is a staging system which is an expression of the anatomical extent of the disease based on the extent of the primary tumor (T), absence or presence of and extent of regional lymph node metastasis (N) and absence or presence of distant metastasis [21]. Gastric cancer was classified macroscopically according to the Borrmann classification system into five types, as follows: Type I (Polypoid/fungating) tumor, Type II (Superficial spreading) tumor, Type III (Ulcerating) tumor, Type IV (Diffusely infiltrating/linitis plastica) tumor and Type V (Unclassified) tumor [22]. The histological classification was based on Laurens (1965) classification as follows: (1) Intestinal type, (2) Diffuse type and (3) Mixed type [23].

The diagnosis of gastric cancer was performed by upper GI endoscopy, double contrast barium meal and laparotomy, and confirmed pathologically by upper GI endoscopic and laparotomy biopsies. Other investigations performed included laboratory studies, such as full blood laboratory studies. Treatment modalities included surgery, chemotherapy and radiotherapy. Patients were followed up for up to five years or death. Survival analysis was carried out with survival defined as the time between the date of commencement of treatment and the date of last follow-up or death. The recurrence of disease was confirmed by physical findings, radiological studies, endoscopic examination with biopsy and surgery.

### Statistical analysis

Data collected were analyzed using SPSS computer software version 17.0 (SPSS, Inc., Chicago, IL, USA). Data were summarized in the form of proportions and frequency tables for categorical variables. Continuous variables were summarized using mean, median, mode and standard deviation. Chi-square test (X^2^-test) was used to test for significance of associations between the predictor and outcome variables in the categorical variables. Student’s *t*-test was used to test for significance of associations between the predictor and outcome variables in the continuous variables. Significance was defined as a *P*-value of <0.05. Multivariate logistic regression analysis was used to determine predictor variables that are associated with outcome.

### Ethical consideration

Ethical approval to conduct the study was sought from the CUHAS-Bugando/BMC joint institutional ethic review committee before the commencement of the study.

## Results

### Study population and socio-demographic data

Out of 5,134 patients who were registered with malignancies at our center during the study period, 256 were cases of gastric cancer. Of these, 24 patients were excluded from the study due to incomplete data. Thus, 232 patients were enrolled in the study representing 4.5% of cases. There were 172 (74.1%) males and 60 (25.9%) females giving a male to female ratio of 2.9:1. The ages ranged from 21 to 85 years with a median age of 52 years. The modal age group was 51 to 60 years accounting for 53.4% of cases (Table 1). Most of patients, 197 (84.9%), had either primary or no formal education and more than three-quarters of them were unemployed.

**Table 1:** Age distribution of patients (N = 232)

**Age in years**	**Number of patients**	**Percentage**
21 to 30	4	1.7
31 to 40	23	9.9
41 to 50	40	17.2
51 to 60	124	53.4
61 to 70	18	7.8
71 to 80	13	5.6
>80	10	4.3
Total	232	100

### Clinical presentation

The duration of symptoms ranged from 2 to 96 months with a median duration of 16 months. The majority of patients presented between 13 to 24 months (Table 2). According to the Uganda Cancer Working Group (Table 3), the majority of patients, 227 (97.8%), had late symptoms (Group II to V). Only five (2.2%) patients had early gastric cancer. Most patients presented with features of advanced disease such as epigastric mass in 162 (69.8%), obstructive symptoms in 184 (79.3%), bloomer shelves/Krukenberg’s tumors in 88 (37.9%) and ascites in 63 (27.2%). Forty-five (19.4%) patients had evidence of upper gastrointestinal bleeding. Associated jaundice was reported in 24 (10.3%) patients. Alcohol consumption and smoking was reported in 179 (77.2%) and 104 (44.8%) patients, respectively. The use of NSAIDs was reported in 95 (40.9%) patients. Most patients (135, 58.2%) had blood group O rhesus positive while 68 (29.3%) had blood group A. The type of blood group was not documented in 23 (9.9%) patients. Eleven (4.7%) patients had associated pre-operative co-morbid medical illnesses, such as hypertension in four patients, diabetic mellitus in three patients, chronic chest infections in three and renal disease in one patient.

**Table 2:** Duration of symptoms among the 232 gastric cancer patients

**Duration of symptoms (in months)**	**Number of patients**	**Percentage**
0 to 6	24	10.3
7 to 12	60	25.9
13 to 24	130	56.0
>24	41	17.7
Total	232	100

**Table 3:** Groups of symptoms of gastric cancer according to the Uganda Cancer Working Group

**Group**	**Description of symptoms**
I	New dyspepsia after 40 years; with indigestion and no past history suggestive of PUD
II	Insidious: tired, weak and 3As- anemia anorexia asthenia
III	Obstruction; dysphagia fullness belching and vomiting
IV	Lump in epigastrium palpable in about 30% of patients with cancer of the stomach.
V	Silent but presents with ascites, jaundice, Krukenberg’s tumors, Trousseaus’ and Trossier’s sign.

Keys: Group I = Early symptoms, Group II to V = Late symptoms.

### Anatomical site, gross appearance, TNM staging, histopathological type/grade and metastasis

The antrum was the most frequent anatomical site involved in 56.5% of cases and the most common macroscopic appearance according to Borrmann classification system was type III (ulcerating tumor) seen in 53.9% of cases. The gastric adenocarcinoma was the most common histopathological type, occurring in 95.1% of cases, and most of the tumors had a well-differentiated grade in 38.8% of cases (Table 4). Mucinous adenocarcinoma was the predominant type seen in 102 (46.2%) patients. This was followed by tubular adenocarcinoma, papillary adenocarcinoma and signet ring adenocarcinoma in 35 (15.8%), 30 (13.6%) and in 20 (9.0%) patients, respectively. Undifferentiated adenocarcinoma was documented in 34 (15.4%) patients. According to Lauren classification of gastric adenocarcinoma, 120 (54.3%) were intestinal, 56 (25.3%) were diffuse, 35 (15.8%) were mixed, and 10 (4.5%) were unclassifiable. According to TNM staging, 92.1% of the patients were diagnosed with advanced gastric cancer (Stages III and IV). Most patients (38.8%) had no evidence of metastases. Lymph node metastasis at the time of diagnosis was recorded in 31.9% of cases. Lymph node involvement was greater in signet ring cell (100%) than in other types of carcinoma. Distant metastasis accounted for 29.3% of cases (Table 4) and occurred mainly to the transverse colon, adnexia, peritoneum and liver.

**Table 4:** Anatomical site, macroscopic appearance, histopathological type/grade and metastasis at the time of diagnosis

**Variables**	**Response**	**Frequency**	**Percentage**
Anatomical site	Cardia	12	5.2
	Fundus	40	17.2
	Body	30	12.9
	Antrum	131	56.5
	Diffuse	19	8.2
Macroscopic appearance (Borrmann’s classification)	Type I (Polypoid/fungating)	68	29.3
	Type II (Superficial spreading)	2	0.9
	Type III (Ulcerating)	125	53.9
	Type IV (Linitis plastica )	10	4.3
	Type V (Unclassified)	7	3.0
	Not documented	20	8.6
Histopathological type	Adenocarcinoma	221	95.3
	Primary Lymphoma	3	1.3
	Gastrointestinal stromal tumors of gastric malignancies	3	1.3
	Others: carcinoid tumors, small cell carcinoma, carcinosarcoma	3	1.3
	Metastatic tumors from breast and malignant melanoma	2	0.8
Histopathological grade	Well differentiated	90	38.8
	Moderately differentiated	76	32.8
	Poorly differentiated	46	19.8
	Not documented	20	8.6
TNM Staging	I	2	0.8
	II	3	1.3
	III	152	65.5
	IV	63	27.2
	Not documented	12	5.2
Metastasis at the time of diagnosis	Lymph node metastasis	74	31.9
	Distant metastasis	68	29.3
	No evidence of metastasis	90	38.8

### Diagnosis of gastric cancer

The diagnosis of gastric cancer was confirmed pathologically by upper GI endoscopic biopsies in 198 (85.3%) patients and the remaining 34 (14.7%) patients were diagnosed during laparotomy for other pathologies, such as gastric perforation in 12 (35.3%) patients, gastric outlet obstruction in 10 (29.4%) patients, obstructive jaundice in 6 (17.6%) patients, upper GI bleeding in 5 (14.7%) patients and an intra-abdominal mass in 3 (8.8%) patients.

### Treatment modalities

Out of 232 patients, 223 (96.1%) patients underwent surgical procedures for gastric cancer and the remaining 9 (3.9%) patients were unfit for surgery. Of the type of surgical procedures performed, gastro-jejunostomy was the most frequent performed surgical procedure, accounting for 53.8% of cases (Table 5). In addition to gastric resection, transverse colonic resection with colo-colonic anastomosis, Krukenberg’s tumors excision and splenectomy were performed in 46 (20.6%), 34 (15.2%) and 3 (1.3%) patients, respectively. Of the 223 patients that underwent surgical intervention, lymphadenoctomy was reported in 64 (28.7%) patients. Of these, 48 (75.0%) had D1 lymphadenoctomy and the remaining 16 (25.0%) had D2 lymphadenoctomy. Five (2.2%) gastrectomies were deemed curative by excising all macroscopic disease leaving histological margins free of tumor (R0). In 144 (64.5%) and 74 (33.3%) patients, there was microscopic (R1) and macroscopic (R2) residual disease respectively.

**Table 5:** Type of surgical procedures performed in 223 patients with gastric cancer

**Type of surgical procedures**	**Frequency**	**Percentage**
Gastro-jejunostomy	120	53.8
Partial distal gastrectomy	52	23.3
Exploratory laparotomy + biopsy	50	22.4
Total gastrectomy	1	0.1
Total	223	100

The use of chemotherapy was documented in 56 (24.1%) patients. Of these, 5 (8.9%) were given chemotherapy as a neo-adjuvant therapy, whereas, in the remaining 51 (91.1%), patients, chemotherapy was used as adjuvant therapy. The 5-fluorouracil (5-FU), either alone or in combination with other cytotoxic agents, such as cyclophosphamide, methotrexate, vincristine and adriamycin, was the most commonly used drug. Radiotherapy was used as adjuvant therapy in only 12 (5.1%) patients.

### Clinical outcome and follow-up of patients

Overall, 198 (88.8%) patients had significantly improved quality of life postoperatively as evidenced by improved Karnofsky performance status (KPS), control of vomiting and reduced pain, and were discharged home. Postoperative complications were reported in 86 (37.1%) patients (Table 6). Forty-two patients died in hospital, giving a mortality rate of 18.1%. According to multivariate logistic regression analysis, preoperative co-morbidity, high histological grade and stage of the tumor, and presence of metastases at the time of diagnosis were the main predictors of death (*P* <0.001). The hospital stay ranged between 2 and 56 days with a median of 16 days. Patients who developed postoperative complications had a longer hospital stay (*P* = 0.013). Follow-up ranged from 2 to 64 months, with a median of 14 months. At the end of five years, only 76 (32.8%) patients were available for follow-up and the remaining 156 (67.2%) patients were lost to follow-up. Only 16 (6.9%) patients were alive up to five years. The overall five-year survival rate for patients with intestinal-type carcinoma was higher than that of patients with diffuse carcinomas (*P* = 0.013). Evidence of cancer recurrence was reported in 45 (19.4%) patients. Positive resection margins, stage of the tumor and presence of metastasis at the time of diagnosis were the main predictors of local recurrence (*P* <0.001).

**Table 6:** Postoperative complications (N = 86)

**Postoperative complications**	**Frequency**	**Percentage**
Wound infection	45	52.3
Pneumonia	23	26.7
Peritonitis	12	14.0
Intra-abdominal abscess	10	11.6
Anastomotic leakage	8	9.3
Urinary tract infection	5	5.8
Intestinal obstruction	4	4.6
Duodenal sump leak	3	3.5
Other complications	5	5.8

## Discussion

In this review, gastric cancer accounted for 4.5% of all histopathologically-diagnosed malignancies seen during the studied period in our setting. These data are comparable with other African studies which reported the incidence of gastric cancer to range from 1.1% to 6.0% of all cancers [6,7,24,25]. High figures of 16.3 and 15.1% of all malignancies for males and females, respectively, were reported by Kitinya *et al.*[8] in northeastern Tanzania. Dietary, genetic factors and variation in the infectivity rate of *H. pylori* may be responsible for this regional variation [7,9]. Our figure for gastric cancer in this study may actually be underestimated by the retrospective nature of the study. A better picture of the incidence of gastric cancer in this region requires a prospective comprehensive data collection.

In agreement with other studies [26,27], the peak age incidence of gastric cancer in this study was found to be in the fifth decade of life, which is about a decade or two earlier compared to the findings in developed countries [7]. It is possible that the earlier age occurrence of gastric cancer is related to the life expectancy in the country, rather than any special demographic feature of gastric cancer. Compared with findings in developed countries, gastric cancer in sub-Saharan Africa tends to be very aggressive with short periods of time between the onset of symptoms and diagnosis [16]. It has been reported that the occurrence of gastric cancer at a young age is associated with a worse prognosis [13,16].

The male predominance demonstrated in this study was in keeping with previous observations reported in studies done elsewhere [26-28]. The exact reason for this male preponderance is not known, although it is possible that estrogen may protect women against the development of this form of cancer [29].

A strong association with socio-economic status (SES) has been frequently observed, with individuals of lower SES having higher risk. SES is, of course, not a causal factor, but is a surrogate for many other factors, including sanitary and dietary conditions [10]. As reported in other studies done in developing countries [6-8], the majority of patients in this study had low socioeconomic status with poor education and more than three-quarters of them were unemployed. This observation has an implication on accessibility to health care facilities and awareness of the disease.

The etiopathogenesis of gastric cancer in developing countries is of great interest. It is possibly multifactorial and associated with complex interactions. It is, however, very difficult to know the precise roles of the different factors, such as genetic, premalignant lesions, *H. pylori* infection and diet [9,24,30]. The association between chronic *H. pylori* infection and the development of gastric cancer remains controversial [31]. Several studies have shown significant associations between *H. pylori* seropositivity and gastric cancer risk [9,24,30,32,33]. It is, however, not known why some individuals with *H. pylori* infection develop gastric cancer whereas others do not. The virulence factors of *H. pylori* have been investigated. There is increasing evidence to suggest that certain *H. pylori*, containing a gene called CagA, associated with cytotoxin expression, are more strongly associated with gastric cancer. Several studies have suggested that *CagA* positive *H. pylori* are more common in patients who develop gastric cancer [32,33]. Gastric cancer is generally accepted as a multistep-progression disease from chronic gastritis, chronic atrophic gastritis, intestinal metaplasia, dysplasia and, subsequently, to cancer. Infection with *H. pylori* has been linked to gastric carcinogenesis [34]. It is the main pathogenic factor in the development of chronic atrophic gastritis and intestinal metaplasia [35]. Determination of *H. pylori* seroprevalence was not performed in this retrospective study, because tests for *H. pylori* status were not routinely performed in patients with gastric cancer during the study period and, therefore, it was difficult to establish the association between *H. pylori* infection and gastric cancer.

Prospective studies have demonstrated a significant dose-dependent relationship between smoking and gastric cancer risk [36,37]. There is little support for an association between alcohol and gastric cancer [38]. In this study, we could not determine the association among gastric cancer and smoking and alcohol.

In the present study, the majority of patients presented late with an advanced stage of cancer (stage III and IV), which is in keeping with other studies in developing countries [6-8]. If gastric cancer is diagnosed at an early stage, patients can have a highly favorable prognosis and avoid extended surgery, which may produce complications, especially in the elderly people. However, early symptoms of gastric cancer are non-specific and vague and, therefore, many people in our area who have dyspeptic symptoms are treated for peptic ulcers regardless of the cause of dyspepsia. Subsequently, some of these patients, whose cause of dyspepsia is cancer, are diagnosed with late-stage gastric cancer or one of its complications. Late presentation in our study may be attributed to lack of awareness of the disease, low standard of education, low socioeconomic status, lack of accessibility to health care facilities and lack of screening programs in this region. As gastric cancer appears to occur at a younger age in our population as compared to the Western world, patients over 40 years of age with vague dyspeptic disorders or a long history of epigastric pain should be recommended for esophagogastroduodenoscopy. If any suspicious lesion is observed, multifocal biopsies should be taken. Of course, in order to achieve this there is a need to have endoscopy equipment available at least at all regional referral hospitals and to train more surgeons and physicians in endoscopy.

This study showed a wide spectrum in the gross and histopathological features. The common anatomical site for gastric cancer in this study was gastric antrum, which is similar to studies done in developing countries [6-8], but at variant with what is obtained in developed countries where gastric cardia is becoming the most common site of gastric cancer [39]. Grossly, according to the Borrmann classification system, the ulcerating type was the most common tumor in this study. Similar macroscopic appearance was reported by Cassell and Robinson [40]. However, our findings did not match with those of Schindler *et al.*[41], who found infiltrative lesion (linitis plastica) to be the most common type. The most common histopathological type of gastric cancer in this study was adenocarcinoma, accounting for 95.1% of cases, which is consistent with what is reported in the literature [6-8,10,20]. Other reported gastric neoplasms are gastric lymphoma, and gastrointestinal stromal types of gastric malignancies, which is similar to the worldwide experience [20]. The differences in prognosis and treatment approaches require the need for distinction and differentiation of tumor types. More than half of the gastric adenocarcinomas in this study were the intestinal type, based on Lauren classification. Compared with the diffuse types, the intestinal type is known to be associated with a better prognosis [25]. The overall five-year survival rate for patients with intestinal-type carcinoma in this study was higher than that of patients with diffuse carcinomas. Similar findings were also reported by Riberio *et al.*[42], who found the five-year survival rate to be higher in patients with intestinal type than diffuse type cancer.

The development of endoscopic techniques has improved the proportion of gastric cancers detected at an early stage, particularly in Japan, which has the highest incidence of the disease and the most developed programs for screening [43]. Upper gastrointestinal endoscopy, which is an important diagnostic tool in patients with gastric cancer, is not widely available and not easily affordable to most low socioeconomic class patients in Tanzania. Early detection of the tumor is usually possible when all dyspeptic patients had upper gastrointestinal endoscopy as may occur in developed countries, such as Japan and the United State of America [24].

Lymph node involvement is one of the most important prognostic factors in gastric cancer [44]. Lymph node metastasis at the time of diagnosis in this study was recorded in 31.9% of cases. Lymph node involvement in our series was greater in signet ring cell than in other types of carcinoma. Similar observation was reported by Gürsan *et al.*[45]. High lymph node metastasis in this study is attributed to the late presentation in the majority of patients and this confirmed the highly metastatic potential of gastric cancer. In the present study, distant metastasis was documented in 29.3% of cases and occurred mainly to the transverse colon, adnexia, peritoneum and the liver. A similar distant metastatic pattern was reported by Alatise *et al.*[46] in Nigeria. Late presentation in our area in the majority of patients may also be responsible for the high distant metastatic rate.

The treatment of gastric cancer requires a multidisciplinary approach. Treatment modalities of gastric cancer include surgery combined with chemotherapy and radiotherapy given either as neo- or adjuvant therapy [20]. Surgery is and, most probably, will remain the cornerstone of curative management of resectable gastric cancer; however, this benefit is limited to patients who present with early and, perhaps, localized disease [46]. Complete resection of a gastric tumor with resection of adjacent lymph node is the only chance for a cure [46,47]. However, most of the patients we see in our environment present late with advanced disease at the time of diagnosis, for which only palliative surgery is possible. In this study, only 2.2% of patients had gastric resection with curative intent, 22.4% had gastrectomy and 53.8% of patients underwent gastro-jejunostomy alone due to the advanced nature of the disease. Only a very small proportion of patients in this study had resectable lesions. The low resection rate of gastric cancer with “curative” intent in this study could be explained by the high proportion of patients with advanced gastric cancer at presentation. Our patients tended to present late as evidenced by the facts that there was a long interval between onset of symptoms and presentation. This is similar to findings in previous studies in other developing African and East European countries [47-49]. While surgical resection remains the cornerstone of gastric cancer treatment, the optimum extent of nodal resection remains controversial, with randomized studies failing to show that the D2 procedure improves survival when compared with D1 dissection [49]. In the present study, D2 lymphadenoctomy was carried out on fit patients with locally advanced disease. However, for optimal postoperative recovery and functional outcome, less radical surgery (D1 lymphadenoctomy) was performed on high risk and very old patients and those with wide-spread metastatic disease. In a D2 resection all tumor and N2 lymph nodes are resected, while in a D1 resection only N1 lymph nodes are removed and in a D0 resection only the tumor is removed without the lymph nodes [49]. The scope of lymphadenectomy should be individualized and decided based on an accurate preoperative and intraoperative assessment of the extent of disease and the patient’s fitness. Patients that are fit for surgery should have a D2 lymphadenectomy with preservation of the pancreas and spleen. For more advanced cases and high risk patients, a more limited D1 lymphadenectomy should be carried out. The high rate of D1 lymphadenoctomy in our series can be explained by the fact that the majority of patients in our environment present late with advanced disease at the time of diagnosis and only palliative surgery was possible.

Multimodal treatment involving chemotherapy or radiotherapy, in addition to surgery, is thought to be a promising strategy for improving loco-regional control of gastric cancer [50]. The majority of gastric cancer patients in this study had stage III or IV disease at presentation and were, therefore, candidates for adjuvant therapy. In our series, the use of chemotherapy and radiotherapy was reported in only 22.1% and 5.1% of cases, respectively. Lack of accessibility to radiotherapy and other adjuvant therapies, such as monoclonal antibodies, angiogenic inhibitors and antisense agents, may be responsible for discouraging results and low use of adjuvant therapy in this study. Many studies reported the vital role played by adjuvant chemo radiotherapy [46-48].

In this study, the postoperative complication rate was 37.1%, which is a higher rate than that reported by other authors [51,52]. Surgical wound infection was the most common complication but was usually superficial and easily controlled by local wound care. Additional operations were required in 15 (6.7%) patients who developed postoperative complications, such as anastomotic leak, intra-abdominal abscesses, peritonitis and intestinal obstruction. These required additional operations that are associated with prolonged hospital stay and the cost of treatment. Improvement of surgical technique is, therefore, crucial to lower the occurrence of these postoperative complications while their prompt recognition and treatment would reduce the attendant mortality. In addition, prior treatment of preoperative comorbidities is essential to the postoperative recovery of patients with gastric cancer. A higher complication rate in this study is attributed to late presentation and delayed definitive treatment. The presence of comorbidities and a poor level of fitness in our patients increase the risk of postoperative complications and lowers patients’ ability to survive major complications when they occur.

Despite considerable improvement in the surgical treatment of gastric cancer, recurrences still constitute the main cause of death in surgical patients [53-55]. Recent series showed overall recurrence rates of 22 to 50% after curative surgery, mostly within two years from the operation [53-56]. In our study, recurrence rate was reported to be 19.4% of cases, attributing this to the presence positive resection margins, the high stage of the tumor and presence of metastasis at the time of diagnosis.

The median duration of hospital stay in our study was 16 days, which is higher than that reported in other studies [51,52]. This can be explained by the high rate of postoperative complications which required additional operations/care and, subsequently, prolonged hospital stay.

The postoperative mortality following gastric cancer surgery is 1.7 to 16% in Western countries [57-59]. Our overall mortality rate in the present study was 18.1%, a figure that is comparable to 18.6% reported by Johnson *et al.*[48] in Ethiopia. In the present study, the mortality rate was significantly high in patients with preoperative co-morbidity, high stage and grade of the tumor and those who had metastases.

The prognosis of gastric cancer has remained poor in most developing countries where most patients are already in an advanced stage of the disease at the time of diagnosis, which has been proven both in the present study and in most studies done in developing countries [6,7,46-48]. However, when it is diagnosed and treated early, gastric cancer is curable as a five-year survival rate of over 90% has been achieved in Japan [13]. In this study, the overall five-year survival rate of 6.9% is significantly low compared to the five-year survival rates of gastric cancer patients ranging from 68 to 92% reported in developed countries and Japan [13,47]. The low overall five-year survival rate in the present study may be explained by the fact that most of our patients generally seek medical attention when the disease has reached an advanced stage. Therefore, diagnosis is made when the chance of a full cure is low. The follow-up of patients in this study was generally poor as more than two-third of patients were lost to follow-up by the end of five years.

The potential limitations of this study included the following: first, the fact that information about some patients was incomplete in view of the retrospective nature of the study might have introduced some bias in our findings. Second, we did not determine the association of *H. pylori* with gastric cancer because of lack of necessary facilities at the study center. Third, this study included patients who were evaluated and treated at a single institution, which may not reflect the whole population in this region, despite the fact that approximately 70% of oncology patients in northwestern Tanzania are managed at our center.

However, despite these limitations, the study has provided local data that can help health care providers in the management of patients with gastric cancer. The challenges identified in the management of gastric cancer in our setting need to be addressed in order to deliver optimal care for these patients.

## Conclusions

Gastric cancer in our environment is not uncommon and shows a trend towards a relative young age at diagnosis and the majority of patients present late with advanced stage cancer. Lack of awareness of the disease, poor accessibility to health care facilities and lack of screening programs in this region may contribute to advanced disease at the time of diagnosis. Poor accessibility to adjuvant therapy in our center has also contributed to poor outcome of treatment of these patients. There is a need for early detection, adequate treatment and proper follow-up to improve treatment outcome.

## Abbreviations

BMC: Bugando Medical Centre; CUHAS-Bugando: the Catholic University of Health and allied Sciences-Bugando; PUD: Peptic ulcer diseases; NSAID: Non-Steroidal Anti-inflammatory Drugs; SES: socio-economic status; TNM: is a cancer staging system that describes the extent of cancer in a patient’s body, T describes the size of the tumor and whether it has invaded nearby tissue, N describes regional lymph nodes that are involved, M describes distant metastasis (spread of cancer from one body part to another).

## Competing interests

The authors declare that they have no competing interests.

## Authors' contributions

JBM conceived the study and participated in the literature search, and writing and editing the manuscript. MDM, MK, PLC, FM, PFR and NM participated in study design, data analysis, and manuscript writing and editing. HJ was involved in study design, data analysis, coordination of manuscript writing and editing, and submission of the article. In addition, MK and HJ did the endoscopic examination, while PFR performed histological examinations JBM and PLC provided surgical treatment to patients and NM provided oncological care to patients. All the authors read and approved the final manuscript.
